# Gene Co-expression Network Analysis Suggests the Existence of Transcriptional Modules Containing a High Proportion of Transcriptionally Differentiated Homoeologs in Hexaploid Wheat

**DOI:** 10.3389/fpls.2018.01163

**Published:** 2018-08-08

**Authors:** Kotaro Takahagi, Komaki Inoue, Keiichi Mochida

**Affiliations:** ^1^Bioproductivity Informatics Research Team, RIKEN Center for Sustainable Resource Science, Yokohama, Japan; ^2^Graduate School of Nanobioscience, Yokohama City University, Yokohama, Japan; ^3^Kihara Institute for Biological Research, Yokohama City University, Yokohama, Japan; ^4^Institute of Plant Science and Resources, Okayama University, Kurashiki, Japan

**Keywords:** allopolyploidization, co-expression gene network, hexaploid wheat, homoeolog, transcriptional module

## Abstract

Genome duplications aid in the formation of novel molecular networks through regulatory differentiation of the duplicated genes and facilitate adaptation to environmental change. Hexaploid wheat, *Triticum aestivum*, contains three homoeologous chromosome sets, the A-, B-, and D-subgenomes, which evolved through interspecific hybridization and subsequent whole-genome duplication. The divergent expression patterns of the homoeologs in hexaploid wheat suggest that they have undergone transcriptional and/or functional differentiation during wheat evolution. However, the distribution of transcriptionally differentiated homoeologs in gene regulatory networks and their related biological functions in hexaploid wheat are still largely unexplored. Therefore, we retrieved 727 publicly available wheat RNA-sequencing (RNA-seq) datasets from various tissues, developmental stages, and conditions, and identified 10,415 expressed homoeologous triplets. Examining the co-expression modules in the wheat transcriptome, we found that 66% of the expressed homoeologous triplets possess all three homoeologs grouped in the same co-expression modules. Among these, 15 triplets contain co-expressed homoeologs with differential expression levels between homoeoalleles across ≥ 95% of the 727 RNA-seq datasets, suggesting a consistent trend of homoeolog expression bias. In addition, we identified 2,831 differentiated homoeologs that showed gene expression patterns that deviated from those of the other two homoeologs. We found that seven co-expression modules contained a high proportion of such differentiated homoeologs, which accounted for ≥ 20% of the genes in each module. We also found that five of the co-expression modules are abundantly composed of genes involved in biological processes such as chloroplast biogenesis, RNA metabolism, putative defense response, putative posttranscriptional modification, and lipid metabolism, thereby suggesting that, the differentiated homoeologs might highly contribute to these biological functions in the gene network of hexaploid wheat.

## Introduction

Interspecific hybridization and polyploidization have played important roles in the evolution and diversification of plants (Soltis and Soltis, 2009; Van de Peer et al., 2009). Allopolyploids are originated from hybridization between different species followed by whole-genome duplication (Ramsey and Schemske, 1998; Comai, 2005). Despite the multiple conditions that need to be met for allopolyploidization to occur, including existing populations of parental lines in the same area, overcoming hybrid incompatibility, gametic non-reduction, and chromosome doubling (Osabe et al., 2012), the occurrence of allopolyploids is widespread in various taxonomic groups in plants (Leitch and Leitch, 2008; Barker et al., 2016). Therefore, it has been hypothesized that allopolyploid species have evolutionary advantages compared to their diploid ancestral species (Wendel, 2000; Doyle et al., 2008).

Improved traits that evolved in allopolyploid plants enhanced their productivity and have contributed to the domestication of many crops (Chen, 2010; Renny-Byfield and Wendel, 2014). For example, the allotetraploid *Arabidopsis suecica* has more vigorous growth and produces more seeds than its ancestral species (Solhaug et al., 2016), whereas the allotetraploid *Coffea arabica* can better adapt to changes in temperature than its diploid ancestors (Combes et al., 2013). In allohexaploid wheat (*Triticum aestivum*), both natural and synthetic plants have higher tolerance to salt stress than their diploid and tetraploid ancestors (Dubcovsky and Dvorak, 2007; Yang et al., 2014). These examples suggest that allopolyploidization often leads to increased productivity through fixation of genomic heterozygosity, which improves environmental fitness and contributes to the habitat expansion of a species.

Allopolyploidization can give rise to transcriptional and/or functional changes in homoeologs (genes that are duplicated due to allopolyploidization) (Mochida et al., 2003; Adams and Wendel, 2005; Moore and Purugganan, 2005). Homoeologs can undergo accelerated evolution due to redundant genetic codes that can evolve new functions without constraints (Kaessmann, 2010; Naseeb et al., 2017). A number of studies have revealed their fates as non-functionalized (loss of function of one of the duplicated genes), subfunctionalized (partitioning of function between duplicated genes), and/or neo-functionalized (diversification of function between the duplicated genes) (Lynch and Conery, 2000; Blanc and Wolfe, 2004; Cusack and Wolfe, 2007). Homoeologs in plants often show different expression patterns across tissues, developmental stages, and conditions, suggesting that they have undergone sub- and/or neofunctionalization (Madlung, 2013). The differential employment of homoeologs through dynamic transcriptional regulation may contribute to the enhanced evolutionarily adaptability of allopolyploid species.

A number of studies based on homoeolog-specific gene expression analysis have reported the evolutionary fates of homoeologs in various allopolyploid plants (Adams, 2007; Hughes et al., 2014; Takahagi et al., 2018). Transcriptome analysis has revealed that the expression of multiple ribosomal protein-coding homoeologs in *Brassica napus* is tissue-dependent (Whittle and Krochko, 2009). An investigation of the relative levels of allelic and homoeologous gene expression in cotton revealed that subfunctionalized genes are mainly expressed in reproductive tissues, and non-functionalized alleles are typically derived from the A-genome, indicating potential genome-of-origin bias for neofunctionalization (Chaudhary et al., 2009). Differentiation of expression patterns of homoeologs in allopolyploid species might effect changes in their gene regulatory networks owing to transcriptional and/or functional divergence. The evolutionary changes in gene regulatory networks are thought to facilitate responses to developmental programs and environmental cues in allopolyploids (Chen and Ni, 2006).

Hexaploid wheat, *Triticum aestivum*, is a widely cultivated allohexaploid crop (2n = 6x = 42, AABBDD) that originated from hybridization between the domesticated allotetraploid *Triticum turgidum* (2n = 4x = 28, AABB) and the diploid goat grass *Aegilops tauschii* (2n = 2x = 14, DD) approximately 10,000 years ago, followed by genome duplication (Matsuoka, 2011; Feldman and Levy, 2012). Pfeifer et al. (2014) generated a co-expression gene network of hexaploid wheat and examined the contribution of expression of each homoeolog. They found that several network modules exhibit unbalanced homoeolog expression, which might be associated with biological functions and tissue types (Pfeifer et al., 2014). Recently, Tanaka et al. (2016) reported homoeolog-specific regulation of the floral MADS-box genes in wheat, and differential expression patterns of homoeologs were consistently observed in both natural and synthetic allohexaploid wheat varieties (Tanaka et al., 2016). Moreover, Powell et al. (2017) demonstrated that the wheat transcriptome has homoeolog expression bias toward the B- and D-subgenomes in response to pathogen infection (Powell et al., 2017). The divergent expression patterns between homoeologs suggest that they have undergone transcriptional and/or functional differentiation. However, the distribution of transcriptionally differentiated homoeologs in gene regulatory networks and their related biological functions in hexaploid wheat are still largely unexplored.

In this study, to elucidate homoeologous networks in hexaploid wheat and to explore their differentiation, we retrieved publicly available RNA-sequencing (RNA-seq) datasets from various tissues, developmental stages, and conditions. We categorized hexaploid wheat genes to construct homoeologous groups and identified expressed homoeologous triplets. We also identified differentiated homoeologs that show gene expression patterns that deviate from those of the other two homoeologs. In addition, we explored gene network modules containing a high proportion of differentiated homoeologs in the transcriptome of hexaploid wheat. We assessed enriched functions in the network modules and discussed the evolution of such network modules resulting from transcriptional differentiation of homoeologs in hexaploid wheat.

## Materials and Methods

### Data and Data Processing

All publicly available wheat transcriptome sequence datasets were retrieved from the NCBI Sequence Read Archive (April 26, 2017)^1^. To adjust the data format, the datasets were screened according to the following criteria: (1) RNA-seq data strictly (i.e., no EST, FL-cDNA, etc.) from *Triticum aestivum* samples, (2) total number of sequence reads ≥ 10,000,000, and (3) an average sequence read length is 70–1000 bases. The RNA-seq datasets presenting the following characteristics were also removed from analyses, as they were considered inappropriate for gene expression profiling: (1) datasets resulting from pooled samples, taken at different time points, (2) datasets obtained from chromosome deletion and chromosome addition lines, and (3) datasets obtained for poorly described methodologies. RNA-seq reads of the screened datasets were trimmed using Trimmomatic (v.0.32) (Bolger et al., 2014) with the following settings: -thread 1 LEADING: 20 TRAILING: 20 SLIDINGWINDOW:4:15 MINLEN: 50. To obtain high-quality sequence datasets, the trimmed datasets were further screened according to the following criteria: (1) ≥ 70% of raw reads are maintained after the trimming step and (2) an average sequence read length is 70–1000 bases after trimming. The trimmed reads obtained after the second screening were mapped to the representative cDNA sequences annotated in the genome assembly of Chinese Spring wheat (International Wheat Genome Sequencing Consortium, 2014) downloaded from the Ensembl (v.35)^2^ using the BWA program (v.0.7.8) (Li and Durbin, 2009) with its mem command. To use datasets with high-quality alignments of the reads, those that were not uniquely mapped and/or not paired mapped were removed from the read alignment datasets using custom Perl scripts. In total, 727 read alignment datasets (**Supplementary Table S1**), for which ≥ 50% of raw reads remained after the read removal step, were subjected to further analysis. The reads per million mapped reads (RPM) values were calculated for all genes in the 727 read alignment datasets. Genes with an RPM ≥ 3 in at least eight datasets (≥ 1% of the 727 RNA-seq datasets) were identified as significantly expressed genes.

### Identification of Homoeologous Groups

**FIGURE 1:**
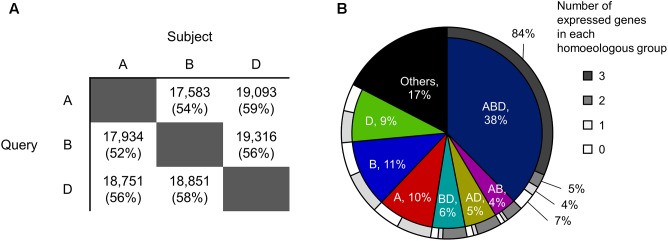
Homoeologous groups in hexaploid wheat. **(A)** Numbers of A-, B-, and D-homoeologs that show high sequence similarity with the other two subgenomes based on BLAST analysis. The e-value cut-off was set at 1e-5 and the sequence identity cut-off was set to 90%. Values in brackets are percentages of the total number of query sequences. **(B)** Proportions of genes classified into each homoeologous group. ABD: sets of three homoeologs that are reciprocal best hits in all pairwise comparisons (i.e., homoeologous triplet); AB, AD, and BD: sets of two homoeologs with reciprocal best hits for two subgenomes and without hits for the other subgenome (i.e., homoeologous doublets); A, B, and D: genes without hits in any of the other two subgenomes (i.e., subgenome-unique genes); Others: genes that are not clustered into an homoeologous groups (e.g., genes with BLAST hits for the other subgenome(s) but that are not reciprocal best hits). The outer circle shows proportions of the number of expressed genes in each homoeologous group.

To identify homoeologous groups, representative protein sequences of the A-, B-, and D-subgenomes annotated in the genome assembly of Chinese Spring wheat (International Wheat Genome Sequencing Consortium, 2014) downloaded from Ensembl (v.35)^2^ were compared against each other using BLASTP (v.2.6) (McGinnis and Madden, 2004), applying an *e*-value cut-off of 1e-5 and a sequence identity cut-off of 90%. Sets of three homoeologs that were reciprocal best hits in all pairwise comparisons were identified as homoeologous triplets (ABD type in **Figure 1B**). Sets of two homoeologs with reciprocal best hits for two subgenomes and without hits for the other subgenome were identified as homoeologous doublets (AB, AD, and BD types in **Figure 1B**). Genes without hits in any of the other two subgenomes were identified as subgenome-unique genes (A, B, and D types in **Figure 1B**).

### t-distributed Stochastic Neighbor Embedding (t-SNE) Analysis

To summarize expression patterns of the genes with an RPM ≥ 3 in a range of 1–7 datasets (spatiotemporally expressed genes), t-SNE analysis was performed using the Rtsne package (v.0.13)^3^ in R (v.3.4.3). The number of iterations was set at 10,000, and parameter theta was set at 0.0.

### Co-expression Network Analysis

To compute co-expression modules of homoeologs, WGCNA analysis (Langfelder and Horvath, 2008) was performed based on the normalized RPM using the one-step automatic network construction method with the following parameters: power = 9, networkType = “signed”, TOMType = “unsigned”, minModuleSize = 30, reassignThreshold = 0, mergeCutHeight = 0.25, numericLabels = TRUE, pamRespectsDendro = FALSE. A soft-thresholding power was selected by evaluating the scale-free topology model fit.

### Identification of Differentially Expressed Genes

For identification of the homoeologous triplets containing co-expressed homoeologs with differential expression levels between homoeoalleles, the gene expression fold changes between homoeologs across the 727 RNA-seq datasets were calculated based on RPM. Pairs of homoeologs with a fold change ≥ 3 and RPM ≥ 3 for at least one of the homoeologs were identified as differentially expressed homoeologs. For the examination of expression bias between homoeologs in the homoeologous triplets, reads used for RPM calculation in a series of RNA-seq datasets (SRR1542404-SRR1542417) (Liu et al., 2015) were subjected to differential gene expression analysis performed by using the edgeR package (v.3.20.9) (Robinson et al., 2010) in R (v.3.4.3). Pairs of homoeologs with a false discovery rate (FDR) ≤ 0.001 and RPM (average of 2 biological replicates in the RNA-seq datasets) ≥ 3 for at least one of the homoeologs were identified as significantly differentially expressed homoeologs.

### Gene Ontology (GO) Enrichment Analysis

The closest homologs of wheat genes in Arabidopsis and rice were identified by BLASTP (v.2.6) (McGinnis and Madden, 2004) searches, applying an *e*-value threshold of ≤ 1e-5. GO terms of the best-hit genes in Arabidopsis and rice were used as the customized annotations for wheat genes. To reduce bias, GO terms that were assigned to more than 5,000 wheat genes were excluded. Enriched GO terms were identified for selected genes using BLAST2GO (v.4.1.9) (Conesa et al., 2005) with the customized annotations of wheat genes. For the estimation of the enriched GO terms of genes that are spatiotemporally expressed (representing genes with an RPM ≥ 3 in less than 1% (eight datasets) of the 727 RNA-seq datasets) or non-significantly expressed (representing genes with an RPM < 3 in all of the 727 RNA-seq datasets), all of the annotated genes in the Chinese Spring wheat chromosomes were used as a reference set. For estimation of the enriched GO terms of the other sets of genes, those in the expressed homoeologous triplets were used as a reference set. The significance threshold was set at FDR ≤ 0.001. The enriched GO terms were summarized based on their semantic similarities using the web-based tool REVIGO^4^ (Supek et al., 2011).

## Results

### Homoeologous Triplets in Hexaploid Wheat

To explore the distribution of transcriptionally differentiated homoeologs in gene regulatory networks and their related biological functions in hexaploid wheat, we identified expressed homoeologous triplets using publicly available RNA-seq datasets. We gathered 727 RNA-seq datasets from hexaploid wheat composed of as many as 517 biosamples relating to various tissues, developmental stages, and conditions, which enabled us to comprehensively explore functional differentiation of transcription regulatory networks in hexaploid wheat (**Supplementary Table S1**). We mapped the quality-checked reads of the RNA-seq datasets to the set of representative cDNA sequences annotated in the genome assembly of Chinese Spring wheat. Using a threshold of RPM ≥ 3 in at least eight datasets (≥1% of the 727 RNA-seq datasets), we found that 73,329 genes (74% of the 99,308 genes corresponding to the representative cDNA sequences assigned to each chromosome) are significantly expressed in hexaploid wheat. To construct putative homoeologous groups, and estimate the number of expressed homoeologs from each homoeoloci, we clustered all the 99,308 genes into 49,710 gene groups based on sequence similarity, using a reciprocal BLAST homology search (**Figure 1A**). Approximately 38% of the genes were classified into gene groups composed of three homoeologs, one from each subgenome (homoeologous triplets, ABD type in **Figure 1B**), in which 84% of the triplets (10,415 triplets) contained three homoeologs significantly expressed in the RNA-seq datasets (expressed homoeologous triplets; **Figure 1B**). We also observed that 31,738 genes (39% of 82,012 genes assigned into each of the homoeologous groups) are expressed from one or two homoeologous loci on the subgenomes, which suggests that approximately 40% of the homoeologous groups contain homoeologs rarely expressed or silenced in the wheat transcriptome (**Figure 1B**).

### Spatiotemporally Expressed Genes in Wheat

**FIGURE 2:**
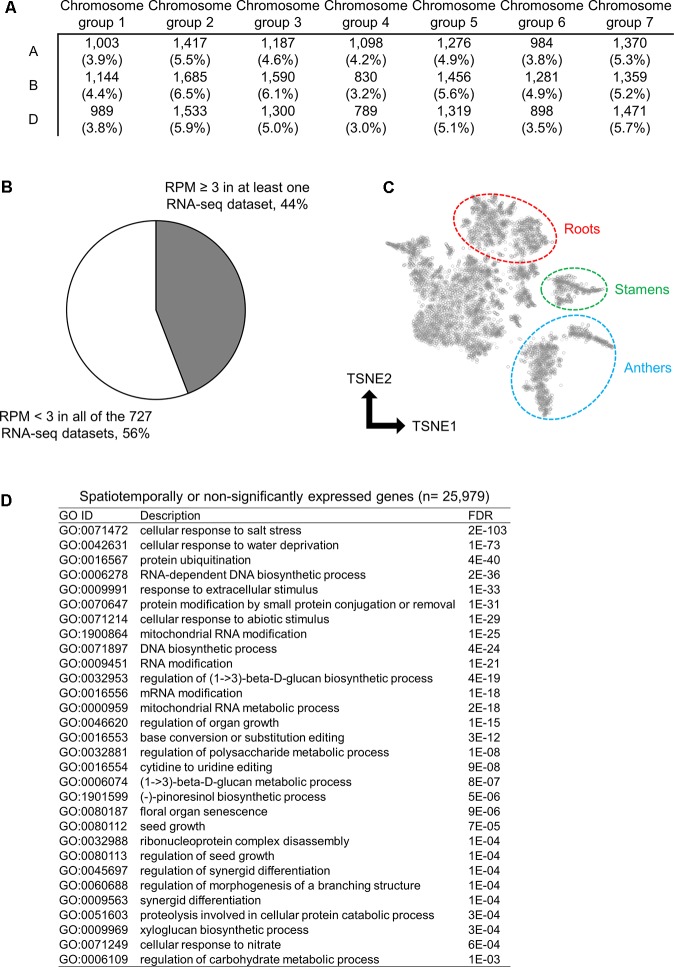
Spatiotemporally or non-significantly expressed genes in hexaploid wheat. **(A)** Distribution of spatiotemporally or non-significantly expressed genes across the 21 wheat chromosomes. **(B)** Proportion of the spatiotemporally expressed genes (RPM ≥ 3 in at least one RNA-seq dataset) and non-significantly expressed genes (RPM < 3 in all of the 727 RNA-seq datasets) in hexaploid wheat. **(C)** t-SNE plot of the spatiotemporally expressed genes. Clusters of genes expressed in roots, stamens, and anthers are circled. **(D)** Enriched GO terms in the biological processes of the spatiotemporally or non-significantly expressed genes in hexaploid wheat.

To characterize the genes found in the wheat transcriptome that are rarely expressed or silenced, we investigated the chromosomal distribution and function of these genes. Using the threshold to identify significantly expressed genes, we classified 25,979 genes as rarely expressed or silenced, which suggested a transcriptional sign of non-functionalization or acceleration of spatiotemporal transcriptional regulation. To further investigate the functional properties of such genes, we assessed their chromosomal distribution; however, no biased distribution of these genes was found across the 21 wheat chromosomes (**Figure 2A**). We found that 44% of the 25,979 genes were expressed in at least one RNA-seq dataset with an RPM ≥ 3, whereas the remaining 56% genes showed an RPM < 3 in all of the RNA-seq datasets, suggesting spatiotemporal expression and insignificant expression, respectively (**Figure 2B**). To summarize the expression patterns of the spatiotemporally expressed genes across the 727 RNA-seq datasets, we clustered and visualized the expression profiles of these genes using the t-SNE algorithm, and detected several clusters corresponding to the RNA-seq datasets from particular tissues, such as roots, stamens, and anthers (**Figure 2C**), suggesting their tissue-specific expressions. To assess gene functions over-represented in the spatiotemporally or non-significantly expressed genes, we performed GO enrichment analysis, and found some enriched GO terms related to the response to abiotic stresses, metabolism, and organ development (**Figure 2D**).

### Expression Bias Between Homoeologs in Hexaploid Wheat

**FIGURE 3:**
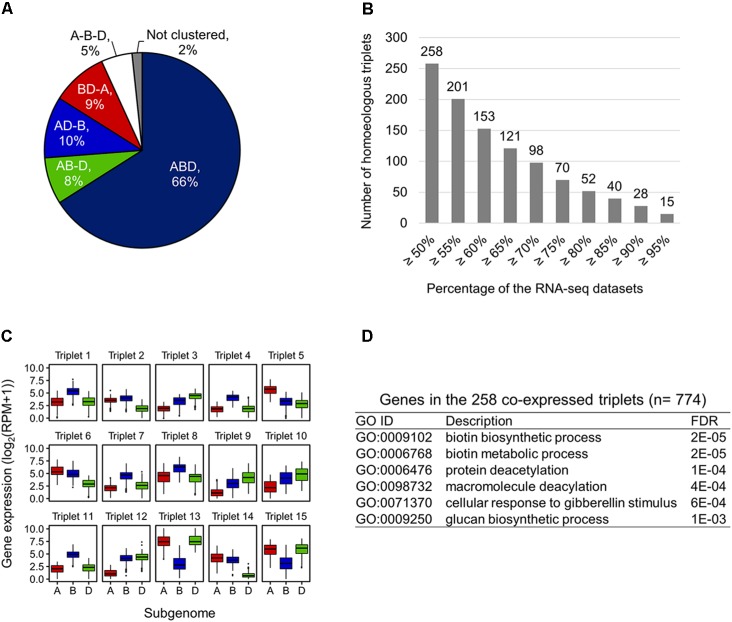
Co-expressed while differentially expressed homoeologs in hexaploid wheat. **(A)** Proportion of co-expression patterns of homoeologs in the expressed homoeologous triplets. ABD, homoeologous triplets in which all three homoeologs are grouped in the same co-expression module; AB-D, homoeologous triplets in which A- and B-homoeologs are grouped in the same co-expression module while D-homoeolog is in another co-expression module; AD-B, homoeologous triplets in which A- and D-homoeologs are grouped in the same co-expression module while B-homoeolog is in another co-expression module; BD-A, homoeologous triplets in which B- and D-homoeologs are grouped in the same co-expression module while A-homoeolog is in another co-expression module; A-B-D, homoeologous triplets in which all three homoeologs are assigned to different modules; Not clustered, homoeologous triplets in which two or all three homoeologs are not assigned to a co-expression module. **(B)** Number of co-expressed triplets containing differentially expressed homoeologs across ≥ 50% of the 727 RNA-seq datasets. **(C)** Box plot of the expression levels of the homoeologs in 15 homoeologous triplets showing a consistent trend of homoeolog expression bias ≥ 95% across the 727 RNA-seq datasets. **(D)** Enriched GO terms in the biological processes of genes in the 258 co-expressed triplets containing differentially expressed homoeologs across ≥ 50% of the 727 RNA-seq datasets.

To examine expression bias between homoeologs in the expressed homoeologous triplets, we computed co-expressed homoeologs and differentially expressed homoeologs based on the 727 RNA-seq datasets. For identification of the co-expressed homoeologs, we applied the WGCNA algorithm, and identified 22 co-expression modules. The results of WGCNA analysis indicated that 66% of the expressed homoeologous triplets possess all three homoeologs grouped in the same co-expression modules (co-expressed triplets, ABD type in **Figure 3A**). For 27% of the triplets, two out of three homoeologs were grouped in the same co-expression modules (AB-D, AD-B, and BD-A types in **Figure 3A**), whereas for the remaining 5% of the triplets, all three homoeologs were assigned to different modules (A-B-D type in **Figure 3A**). To further identify homoeologs that are co-expressed while differentially expressed (representing similar expression patterns across the 727 RNA-seq datasets and differential expression levels between homoeoalleles), we identified differentially expressed homoeologs (fold-change ≥ 3) in the co-expressed triplets, and found that at least 258 triplets contained co-expressed homoeologs with differential expression levels between homoeoalleles across ≥ 50% of the 727 RNA-seq datasets (**Figure 3B**). We also found that 15 co-expressed triplets contained such homoeologs observed in ≥ 95% of the datasets, suggesting a consistent trend of homoeolog expression bias (**Figures 3B,C**). On the basis of our GO enrichment analysis of these genes, we observed several over-represented functions, such as biotin metabolism, protein modifications, and response to gibberellin stimulus (**Figure 3D**). Moreover, to illuminate homoeolog-specific expression patterns relative to particular tissue type that are supported statistically, we examined the expression bias between homoeologs in the homoeologous triplets in a series of RNA-seq datasets related to multiple abiotic stress conditions such as drought, heat, and combined heat and drought (SRR1542404-SRR1542417) (Liu et al., 2015), and found that an increased number of homoeologous triplets contained differentially expressed homoeologs (FDR ≤ 0.001) in response to the drought and heat stress conditions, thereby suggesting the differentiation of transcriptional responsiveness between homoeologs to environmental stresses (**Supplementary Table S2**).

### Transcriptional Modules Containing a Number of Differentiated Homoeologs

**FIGURE 4:**
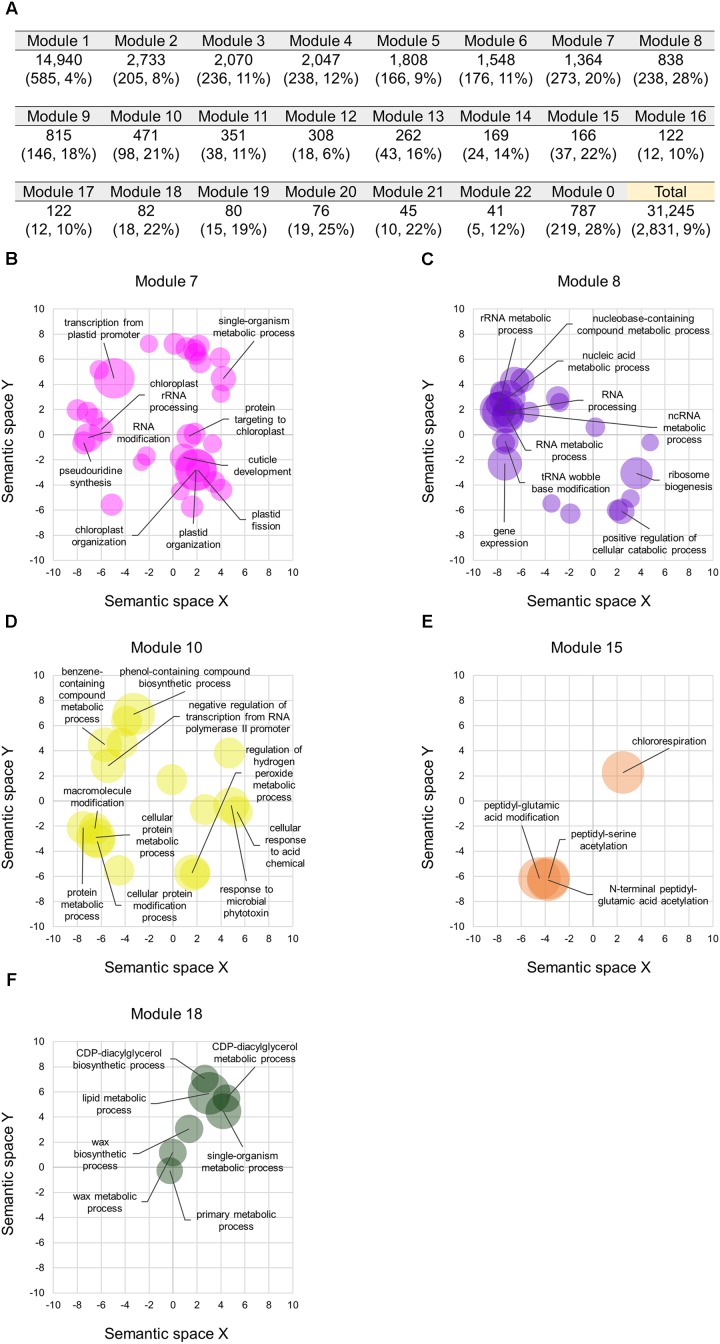
Co-expression modules containing the differentiated homoeologs in hexaploid wheat. **(A)** Number of homoeologs and differentiated homoeologs (numbers with their percentage in brackets) in each of the co-expression modules. The Module 0 represents genes that are not clustered in a co-expression module. Percentages represent proportions of the differentiated homoeologs in each of the co-expression modules. **(B–E)** Enriched GO terms in the biological processes of genes in the co-expression module 7 **(B)**, 8 **(C)**, 10 **(D)**, 15 **(E)**, and 18 **(F)** projected to a 2D semantic space. Circle size represents the –log10 of FDR values calculated using REVIGO analysis. The top ten enriched GO terms are labeled in the plots.

We constructed co-expression gene networks based on the 727 RNA-seq datasets, and thus found that differentiated homoeologs were unevenly distributed in each of the co-expression modules and that several modules contained high proportions of differentiated homoeologs. On the basis of co-expression modules established from our WGCNA analysis, we identified 2,831 homoeologous triplets containing one homoeolog for which the expression pattern deviated from those of the other two homoeologs, which consisted of 9, 10, and 8% of differentiated homoeologs located in A-, B-, and D-subgenomes, respectively (BD-A, AD-B, and AB-D types, respectively, in **Figure 3A**). We also found that such differentiated homoeologs accounted for approximately 9% of all genes used for the WGCNA analysis (10,415 homoeologous triplets; 31,245 genes), whereas seven co-expression modules contained a high proportion of differentiated homoeologs, accounting for ≥ 20% of the genes in each module (**Figure 4A**). To estimate enriched biological functions for the genes within the co-expression modules containing a number of differentiated homoeologs, we performed GO enrichment analysis, and found that five of the co-expression modules are abundant in genes involved in biological processes such as chloroplast biogenesis (module 7; **Figure 4B**), RNA metabolism (module 8; **Figure 4C**), putative defense response (module 10; **Figure 4D**), putative posttranscriptional modification (module 15; **Figure 4E**), and lipid metabolism (module 18; **Figure 4F**). These findings suggest that differentiated homoeologs might highly contribute to these biological functions in the gene network of hexaploid wheat.

## Discussion

Through our homoeologous gene expression analysis of hexaploid wheat based on a number of RNA-seq datasets, we demonstrated a landscape of transcriptional differentiation among homoeologs. Our comprehensive list of genes that were significantly expressed from one or two homoeologous loci enabled us to identify those genes that may have undergone transcriptional suppression or be directed to spatiotemporal expression. Leach et al. (2014) reported that 55% of genes in hexaploid wheat are expressed from one or two homoeologous loci on the subgenomes in root and shoot tissues (Leach et al., 2014). Using the RNA-seq datasets of 90 wheat lines, Wang et al. (2017) found that approximately 60% of wheat genes are expressed from one or two homoeologous loci in reproductive tissues (Wang et al., 2017). Our findings based on more comprehensive transcriptome datasets showed that, compared with previous observations, a smaller number of genes (∼40% of genes assigned into each of the homoeologous groups) are expressed from one or two homoeologous loci (**Figure 1**). These observations suggest that approximately 15–20% of wheat genes, including the silenced loci considered in previous studies, may contain homoeologs that can be expressed in specific tissues, at different developmental stages, or under different conditions. Our list of the spatiotemporally expressed and non-significantly expressed genes represent as many as 44% of those genes expressed (RPM ≥ 3) in 1–7 datasets out of the 727 RNA-seq datasets, and suggested that some of these are particularly expressed in specific tissues such as roots, stamens, and anthers (**Figures 2B,C**). Although we used a threshold of RPM ≥ 3 in less than 1% (eight datasets) of the 727 RNA-seq datasets to identify spatiotemporally or non-significantly expressed genes, this threshold depends on the proportion of samples from similar tissues in the dataset, which might present genes specifically expressed in unusually sequenced samples. To further explore spatiotemporally expressed genes, transcriptome datasets obtained from anatomically- or seasonally-distinct samples should be analyzed using emerged technologies such as laser-capture microdissection RNA-seq (LCM RNA-seq) (Zhan et al., 2015) and field transcriptome sequencing (Plessis et al., 2015). These findings may suggest that such genes expressed only from one or two homoeoalleles undergo transcriptional silencing, probably through differentiation of expression patterns and specialization of spatial expression. Consequently, such duplicated genes might be non-functionalized through promoter malfunctions or repression of other transcriptional machineries as a process of functional diploidization (Levy and Feldman, 2002; Rajkov et al., 2014).

Our gene co-expression network analysis enabled us to identify homoeologous triplets containing homoeologs that are co-expressed while differentially expressed (2.5% of the 10,415 expressed homoeologous triplets), as well as differentiated homoeologs that are classified into co-expression modules that differ from the other two homoeologs (27% of the 10,415 expressed homoeologous triplets) (**Figures 3A,B**). The results of our comprehensive analysis provide evidence that may suggest that most of the differential expression observed between homoeologs represents an alteration of expression patterns in hexaploid wheat. The results of our co-expressed gene network analysis enable us to identify transcriptional modules that contain abundant differentiated homoeologs involved in several particular biological processes, which might have evolved such biological functions in hexaploid wheat through its allopolyploidization (Chen et al., 2007; Feldman and Levy, 2012). Multiple studies have provided evidence to suggest that homoeolog subfunctionalization may be related to enhanced adaptability to adverse environmental conditions in various allopolyploid species, such as tetraploid cotton, tetraploid coffee, and hexaploid wheat (Liu and Adams, 2007; Hu et al., 2011; de Carvalho et al., 2014; Liu et al., 2015). Consequently, our results suggest that along with other genes, such differentiated homoeologs may have innovated transcriptional networks, which may have contributed to adaptation to environmental change as well as to enhanced productivity during the evolution of hexaploid wheat.

The large number of RNA-seq datasets analyzed in the current study allowed integrating the transcriptional properties of each homoeologous triplet into a dataset (**Supplementary Table S3**), thereby providing a useful information resource for understanding the evolution and function of duplicated genes in hexaploid wheat. Moreover, our analyses using the datasets enabled us to demonstrate the presence of co-expression modules containing a high proportion of differentiated homoeologs in hexaploid wheat, which in turn allowed us to dissect its complex transcriptome derived from duplicated genomes. The considerable recent advances in whole-genome assembly in Triticeae species, including hexaploid wheat and its ancestors (Ling et al., 2013; Mochida and Shinozaki, 2013; International Wheat Genome Sequencing Consortium, 2014; Luo et al., 2017), provide us with an opportunity to further explore sub-/neofunctionalized homoeologs and elucidate the diploidization process that occurred during the evolution of hexaploid wheat after allopolyploidization. Such analysis will enable us to identify genes and transcriptional modules that may be associated with adaptive traits in hexaploid wheat. Such genes and transcriptional modules might also prove useful in enhancing the adaptation of staple crops to counter the potentially adverse impacts of global climate changes and improve their productivity.

## Author Contributions

KT and KM designed the work. KT and KI performed the bioinformatics analysis. KT and KM wrote the manuscript.

## Conflict of Interest Statement

The authors declare that the research was conducted in the absence of any commercial or financial relationships that could be construed as a potential conflict of interest.

## Footnotes

1 https://www.ncbi.nlm.nih.gov/sra

2 http://plants.ensembl.org/Triticum_aestivum/Info/Index

3 https://github.com/jkrijthe/Rtsne

4 http://revigo.irb.hr/
